# Nirmatrelvir/ritonavir in heart transplant recipients with COVID-19: A case report on pharmaceutical care and drug interaction management

**DOI:** 10.1097/MD.0000000000043122

**Published:** 2025-06-27

**Authors:** Jing Zhang, Jun Su, Xiongjie Yu, Wei Yue, Jinhua Chen, Lu Xu

**Affiliations:** a Pharmacy, Wuhan Asian Heart Hospital, Wuhan, China; b School of Life Sciences, East China Normal University, Shanghai, China.

**Keywords:** COVID-19, heart transplantation, nirmatrelvir/ritonavir, pharmaceutical care

## Abstract

**Rationale:**

To evaluate the effect of pharmaceutical care provided by clinical pharmacists during COVID-19 treated with nirmatrelvir/ritonavir in heart transplant (HT) patients.

**Patient concerns:**

Three HT recipients were admitted to the hospital after testing positive for severe acute respiratory syndrome coronavirus-2 (SARS-CoV-2) nucleic acid. Due to factors such as underlying immunosuppression resulting from the heart transplantation and the use of antirejection medications, patients who have undergone heart transplantation and become infected with SARS-CoV-2 exhibit significant differences in pathological responses and drug metabolism in the body compared to other patients. During the treatment period with nirmatrelvir/ritonavir, clinical pharmacists provided comprehensive pharmaceutical care for these patients throughout the course.

**Diagnoses:**

After heart transplantation, testing positive for SARS-CoV-2 nucleic acid.

**Interventions:**

During the treatment of the 3 patients with nirmatrelvir/ritonavir for COVID-19, clinical pharmacists provided pharmaceutical care from aspects such as medication indications, dosage administration, drug adjustments, drug interactions, and adverse reaction monitoring, in order to develop individualized medication regimens for the patients.

**Outcomes:**

Clinical pharmacists actively predicted, identified, and resolved issues that arose during the treatment of HT recipients with nirmatrelvir/ritonavir. They monitored the blood concentration of tacrolimus and the interactions between nirmatrelvir/ritonavir and other medications throughout the treatment process. All 3 patients were cured and discharged from the hospital.

**Lessons:**

During the treatment process, managing complex polypharmacy posed significant challenges. Clinical pharmacists implemented individualized therapeutic drug monitoring to optimize antiviral efficacy. Through rational and effective pharmaceutical care, the clinical cure rate for HT recipients infected with COVID-19 was improved.

## Graphical abstract

**Figure:**
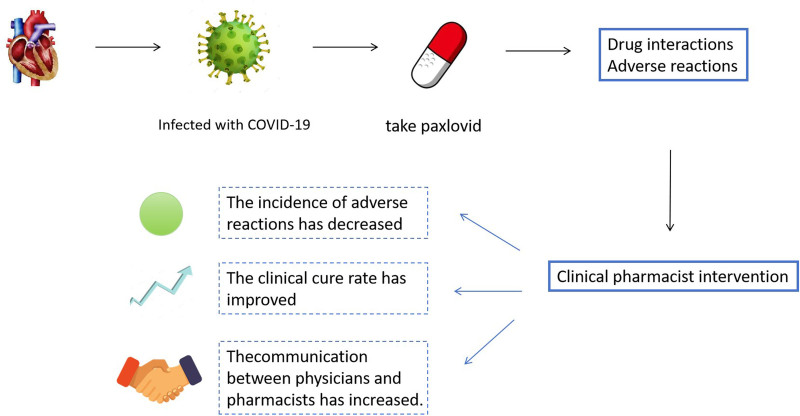

## 1. Introduction

Since the outbreak of novel coronavirus (severe acute respiratory syndrome coronavirus-2 [SARS-CoV-2]) infection in late 2019, the predominant variant circulating globally has become the 5th-generation Omicron variant.^[1]^ On November 22, 2021, the U.S. Food and Drug Administration granted emergency use authorization for the new drug nirmatrelvir tablets/ritonavir tablets co-packaged, to be marketed. On February 12, 2022, the National Medical Products Administration approved the import registration certificate for nirmatrelvir/ritonavir in accordance with the special drug approval procedure. Its core component nematrelvir is an inhibitor of the SARS-CoV-2 3CL protease (Mpro), which blocks viral replication. Ritonavir inhibits CYP3A-mediated metabolism or breakdown of nirmatrelvir, allowing nirmatrelvir to maintain an effective concentration in the body for a longer period of time,^[2,3]^ it is used for the treatment of mild to moderate-risk adults and children aged ≥ 12 years with a body weight ≥ 40 kg, as well as individuals at high risk of severe illness who are infected with SARS-CoV-2.^[4]^

The COVID-19 pandemic has posed unique challenges to solid organ transplant recipients, who face elevated risks of severe infection and mortality due to lifelong immunosuppression. Heart transplant (HT) recipients are particularly vulnerable, with reported.^[1]^ COVID-19-related mortality rates ranging from 20% to 30% in early studies (significantly higher than in the general population). Patients after heart transplantation face significant differences in pathological responses and drug metabolism compared with other patients due to their underlying low immunity and long-term use of immunosuppressants, leading to complex multimedication management during the treatment process.^[5]^ Critically, drug–drug interactions (DDIs) exacerbate these risks, as HT recipients require complex polypharmacy regimens, including immunosuppressants (e.g., tacrolimus, cyclosporine), cardiovascular medications (e.g., beta-blockers, statins), and antiviral therapies (e.g., nirmatrelvir/ritonavir). This article describes the entire process of clinical pharmacists’ involvement in the treatment of 3 HT patients with COVID-19 via nirmatrelvir/ritonavir. All 3 patients recovered and were discharged from the hospital after receiving medical treatment. Based on the Diagnosis and Treatment Protocol for COVID-19 (9th Trial Edition),^[6]^ the Expert Consensus on the Diagnosis and Treatment of COVID-19 in Solid Organ Transplant Recipients (2023 Edition),^[7]^ and the Recommendations for the Use of Inpatient Therapeutic Drugs for COVID-19 in Organ Transplant Recipients (2023 Edition),^[8]^ clinical pharmacists have outlined the key points of pharmaceutical care in the clinical treatment of HT patients, including the selection of dosing regimens, adjustment of dosage and administration, evaluation of drug interactions, and monitoring of adverse reactions, providing a reference for rational drug use in clinical practice.

## 2. General information and pharmacological treatment

### 2.1. Case 1

The patient is a 65-year-old male who underwent heart transplantation on June 2022. On December 26, 2022, he was admitted to the hospital due to “cough, fatigue, and positive COVID-19 nucleic acid test.” Upon admission, his temperature (T) was 38.5 °C, pulse (P) was 86 beats/min, respiratory rate (R) was 21 breaths/min, and blood pressure (BP) was 139/89 mm Hg. His percutaneous arterial oxygen saturation (SpO_2_) was 96%, white blood cell count (WBC) was 2.6 × 10^9^/L, neutrophil percentage (N%) was 76%, hemoglobin was 10^9^ g/L, high-sensitivity C-reactive protein (hs-CRP) was 65.75 mg/L, and B-type natriuretic peptide precursor was 1683 pg/mL. Coarse breath sounds were heard in both lungs. It was the third day of COVID-19 symptoms, and there were indications for the use of nirmatrelvir/ritonavir. On December 27, the patient discontinued the immunosuppressants tacrolimus and mycophenolate mofetil. On December 30, nirmatrelvir/ritonavir (nirmatrelvir 300 mg/ritonavir 100 mg, q12h) was administered for a 5-day course of antiviral treatment. The patient had severe lung infection and was treated with methylprednisolone (40 mg, qd, iv) and piperacillin–tazobactam sodium (4.5 g, q8h, ivgtt) for anti-infection therapy and was also given gamma globulin (200 mg, qd, iv) and sivelestat sodium (0.3 g, qd, iv) to enhance immunity. On January 2nd, the patient experienced gastric discomfort and vomiting, and metoclopramide (20 mg, bid, iv) was added to the treatment regimen. On January 3rd, after the completion of nirmatrelvir/ritonavir treatment, mycophenolate mofetil (0.25 g, q12h) was initiated, and the methylprednisolone injection was switched to oral prednisone tablets (10 mg, qd). A review of the infection indicators revealed a WBC of 7.8 × 10^9^·L^‐1^, N% 90%, and hs-CRP 27.17 mg·L^‐1^. A lung CT scan revealed slight improvement in the infection rate. On January 6th, the dose of mycophenolate mofetil was increased (0.5 g, q12h), and tacrolimus (1 mg, q12h) was added. The patient had a normal temperature, and his mental and physical condition significantly improved. The reviewed tacrolimus concentration was 8.13 ng·mL^‐1^, which was within the normal range, and the patient was discharged on January 10th.

### 2.2. Case 2

The patient is a 66-year-old female who underwent heart transplantation on March 2021. On December 31, 2022, she was admitted to the hospital due to “fever, nausea, vomiting, and diarrhea.” Upon admission, T 36.8 °C, P 98/min, R 20/min, BP 121/72 mm Hg, WBC 0.9 × 10^9^/L, red blood cell count 2.8 × 10^12^/L, N% 45%, hs-CRP 7.60 mg/L, platelet (PLT) 74 × 10^9^/L, glomerular filtration rate (eGFR) 30 mL/min, B-type natriuretic peptide precursor 816.1 pg/mL, urinary protein 1+, and COVID-19 nucleic acid tests were positive, and scattered moist rales were audible in both lower lungs. The patient was immediately given ceftazidime (1 g, q8h, ivgtt) for anti-infective treatment upon admission, and Diyu Shengbai Tablets and Leukogen Tablets were administered to increase the white blood cell count. On January 1st, the reviewed WBC count was still low at 1.4 × 10^9^/L. The clinical pharmacist recommended administering recombinant human granulocyte colony-stimulating factor (100 µg subcutaneous injection) and gamma globulin (200 mg, qd, iv) to accelerate the increase in white blood cells and to discontinue cyclosporine. The lipid-lowering medication was switched from atorvastatin calcium tablets (20 mg, HS) to pitavastatin calcium tablets (2 mg, HS). On January 3rd, nirmatrelvir/ritonavir (nirmatrelvir 150 mg/ritonavir 100 mg, q12h) was initiated for antiviral treatment. The next day, the patient’s renal function indicators further deteriorated (eGFR 24 mL/min). Clinical pharmacists recommended reducing the nirmatrelvir/ritonavir dose to 150 mg/ritonavir 100 mg, qd) and continuing the treatment for 5 days. On January 7th, a review of the lung CT scan revealed improvement in the number of infectious lesions in both lungs compared with before. Mycophenolate mofetil (0.25 g, q12h) was added on 11th January, and cyclosporine capsules (50 mg, q12h) were added on 13th January. On January 16th, the peak concentration of cyclosporine was 501.8 ng/mL and the trough concentration was 49.2 ng/mL, both of which are within the normal range. On January 7, the patient experienced intermittent abdominal pain and was given metoclopramide (10 mg, iv). The next day, the patient’s abdominal pain persisted after eating. The clinical pharmacists recommended the addition of esomeprazole sodium (40 mg, q12h, iv), Bifidobacterium lactobacillus triple viable bacteria tablets (1.5 g, tid), and cefoperazone-sulbactam sodium (1.5 g, q8h, iv) to the treatment regimen. The patient’s abdominal pain significantly improved. On January 17, the review of the COVID-19 nucleic acid test was negative, and the lung CT scan revealed a reduction in the range of infectious lesions in both lungs. There were no chills or fevers for the past 7 days, and her pulse oximetry was above 95%. The patient was ready for discharge.

### 2.3. Case 3

The patient is a 22-year-old male who was admitted to the hospital due to “enlarged heart for 5 years.” He underwent heart transplantation on December 7, 2022. On December 27, the patient experienced headache and fatigue, with T 37.6 ℃, WBC 7.8 × 10^9^/L, N% 80%, hs-CRP 15.74 mg/L, NT-pro BNP 2082 pg/mL. The COVID-19 nucleic acid test was positive. This patient was only 20 days after heart transplantation, and nirmatrelvir/ritonavir should be used early to avoid progression to severe COVID-19. The HT medications tacrolimus (0.5 mg, q12h) and mycophenolate mofetil (0.5 g, q12h) were adjusted to the minimum dosage for administration. Three days later, tacrolimus was discontinued, and nirmatrelvir/ritonavir (nirmatrelvir 300 mg/ritonavir 100 mg, q12h) was administered for antiviral treatment. To avoid drug interactions, amlodipine besylate tablets were replaced with benidipine hydrochloride (5 mg, qd). During the treatment, the patient experienced irritability and poor sleep, so zolpidem tartrate tablets were added (5 mg, qn). On the 4th day of treatment, the COVID-19 nucleic acid test was negative, and the lung CT showed improvement compared to before. Therefore, nirmatrelvir/ritonavir was discontinued, and tacrolimus (1 mg, q12h) was initiated. On January 3rd, the trough concentration of tacrolimus was 3.35 ng/mL, which was low. Therefore, the dosage of tacrolimus was increased (2 mg, q12h). On January 6th, the trough concentration of tacrolimus was still low at 2.51 ng.mL^-1^. Genetic testing revealed that the patient’s tacrolimus genotype was CYP3A5*1/*1, which is a fast metabolic type, and clinical pharmacists recommended to use cyclosporin (175 mg, q12h) for antirejection treatment. On January 11th, the peak concentration of cyclosporine was 838.6 ng/mL^,^ and the trough concentration was 137.6 ng/mL, both of which are within the normal range. The patient is in good condition and can be discharged.

The general status of the 3 patients treated with nirmatrelvir/ritonavir are shown in Table 1.

**Table 1:** General status of the 3 patients treated with paxlovid.

	Case 1	Case 2	Case 3
Gender and age	Male, 65 years old	Female, 66 years old	Male, 22 years old
Length of hospital stay	December 26, 2022–January 10, 2023	December 31, 2022–January 17, 2023	November 2, 2022–January 11, 2023
Time of heart transplantation	June, 2022	March, 2021	December 7, 2022
Past medical history	Hypertension for 20 years, and chronic atrophic gastritis	Chronic cholecystitis, splenic infarction, renal infarction, and chronic kidney dysfunction	Heart enlargement for 5 years
Lung CT scan	Segmental atelectasis of the left lower lobe with bilateral lung infection.	Infectious lesions in the middle and lower lobe and left lower lobe of the right lung	A small amount of inflammatory lesions in the lower lobes of both lungs.
Echocardiography	Enlargement of the left atrium, widening of the ascending aorta; thickening of the interventricular septum and left ventricular wall; impaired left ventricular diastolic function.	Slightly thickened interventricular septum, mild tricuspid regurgitation, and impaired left ventricular diastolic function.	Cardiac enlargement
Usage of paxlovid	December 30, 2022 to January 03, 2023, nirmatrelvir 300 mg/ritonavir 100 mg, q12h	On January 4, 2023 nirmatrelvir 150 mg/ritonavir 100 mg, q12h January 5–January 8, 2023 nirmatrelvir 150 mg/ritonavir 100 mg, qd	27 December to December 30, 2022 nirmatrelvir 300 mg/ritonavir 100 mg, q12h
Usage of immunosuppressants	Tacrolimus and mycophenolate mofetil were discontinued on December 27, 2022 Mycophenolate mofetil(0.25 g, q12h) was started on January 3, 2023; mycophenolate mofetil (0.5 g, q12h) + Tacrolimus (1 mg, q12h) were used on January 6	Cyclosporine and mycophenolate mofetil were discontinued on January 1, 2023 Mycophenolate mofetil(0.25 g, q12h) was started on January 11, 2023; cyclosporine capsules (50 mg, q12h) was added on January 13	Tacrolimus was discontinued on December 27, 2022 and mycophenolate mofetil (0.25 g, q12h) was not discontinued. Tacrolimus (1 mg, q12 h) was started on December 30 and (2 mg, q12h) on January 3, 2023; it was switched to cyclosporine (175 mg, Q12h) on January 6
Key points of pharmaceutical care	The drug–drug interaction between Paxlovid and tacrolimus	The patient had low WBC, renal insufficiency, and the drug–drug interaction between Paxlovid and cyclosporine as well as atorvastatin calcium	Short duration after heart transplantation, continued use of low-dose immunosuppressants, rapid metabolizer genotype for tacrolimus, drug–drug interaction between paxlovid and amlodipine besylate, and use of sedative medications.
Adverse drug reactions.	Stomach upset, vomiting	Abdominal pain	None

WBC = white blood cell count.

## 3. Pharmaceutical care

### 3.1. Case 1

An elderly male patient who has undergone heart transplantation and developed a pulmonary infection, with a high risk of severe COVID-19, should be treated with nirmatrelvir/ritonavir as early as possible. The patient had been taking tacrolimus and mycophenolate mofetil to prevent rejection since HT surgery. Tacrolimus is primarily metabolized by CYP3A4,^[9]^ and ritonavir, a component of nirmatrelvir/ritonavir, acts as a strong inhibitor of CYP3A, which can lead to an increase in the blood concentration of tacrolimus and elevate its toxicity level.^[2]^ The mycophenolic acid derivative mycophenolate mofetil, although having no interaction with nirmatrelvir/ritonavir, has been found in some studies to be associated with the occurrence of severe or critical COVID-19.^[10]^ Based on guidelines and the package insert,^[4,8]^ clinical pharmacists recommend that the patient discontinues immunosuppressants 3 days before starting nirmatrelvir/ritonavir treatment. After nirmatrelvir/ritonavir treatment is complete, a low dose of mycophenolate mofetil should be added immediately, and tacrolimus should be added on the third day after completion, with monitoring of blood drug concentrations. Prednisone, a routine medication after HT surgery, is converted to the active metabolite prednisolone in the body, which is metabolized by CYP3A4 and, in combination with nirmatrelvir/ritonavir may increase the exposure to prednisolone. Clinical pharmacists recommend the use of intravenous methylprednisolone during nirmatrelvir/ritonavir treatment to reduce drug interactions and achieve a stronger anti-inflammatory effect. During the treatment, the patient experienced gastric discomfort, and the clinical pharmacist analyzed that it might be due to the adverse drug reaction of nirmatrelvir/ritonavir or the progression of the disease and the vomiting was relieved after symptomatic antispasmodic treatment.

### 3.2. Case 2

The patient’s WBC was 0.9 × 10^9^/L upon admission to the hospital, and oral medication to increase the white blood cell count had little effect. The clinical pharmacist considered that the decreased counts of the 3 blood cell lines might be due to long-term use of immunosuppressants or infection and recommended immediate intravenous infusion of recombinant human granulocyte colony-stimulating factor and gamma globulin to accelerate the increase of white blood cells and enhance the patient’s nonspecific immunity. The patient had chronic renal insufficiency at the time of admission, and the urine protein was 1+. The Nirmatrelvir/Ritonavir package insert recommends that for patients with moderate renal impairment (eGFR 30–60 mL/min), the dosage should be 150 mg of nirmatrelvir tablet and 100 mg of ritonavir tablet, bid; it is not recommended for patients with severe renal impairment (eGFR < 30 mL/min).^[4]^ The patient’s renal function further deteriorated on the second day of treatment (eGFR 24 mL/min). After the patient’s benefit/risk profile was assessed, the physician and clinical pharmacist decided to adjust the dosage to 150 mg of nirmatrelvir tablet and 100 mg of ritonavir tablet, qd, and to continuously monitor the patient’s renal function. On January 7th, the patient’s COVID-19 nucleic acid test result was negative, and the rechecked eGFR was 30 mL/min, indicating that the treatment plan was effective. The clinical pharmacist analyzed that the course of COVID-19 may have a negative impact on patients’ renal function. In addition to considering medication indications and drug interactions, attention should also be given to contraindications that may arise due to changes in the patient’s condition, and close monitoring of dosage, duration of treatment, and changes in renal function indicators should be implemented.

The patient had been taking cyclosporine and mycophenolate mofetil before admission to the hospital. Cyclosporine is metabolized primarily by CYP3A4 and CYP3A5 in the intestine and liver, and concomitant use of nirmatrelvir/ritonavir may increase its plasma concentration, thereby increasing its toxicity level.^[2]^ The clinical pharmacist recommends discontinuing cyclosporine for 3 days before initiating nirmatrelvir/ritonavir treatment, reintroducing cyclosporine on the third day after completing nirmatrelvir/ritonavir treatment, and monitoring of blood drug concentrations should be conducted concurrently. The patient had a history of chronic cholecystitis and experienced abdominal pain during the COVID-19 treatment period. The clinical pharmacist observed that the patient’s abdominal pain was related to eating, suggesting that the patient may have developed intestinal dysbiosis and concurrent peptic ulcers. Therefore, for COVID-19 patients with a weaker digestive system, the clinical pharmacist recommends the prophylactic administration of probiotics to prevent gastrointestinal infections. This can also alleviate symptoms of diarrhea and bloating caused by nirmatrelvir/ritonavir^[7]^ and play an important role in maintaining the homeostasis of the immune system.^[11–13]^ The patient had been taking atorvastatin calcium tablets to regulate blood lipids, which are metabolized by CYP3A. The clinical pharmacist suggested replacing it with pitavastatin, which is metabolized by UGTs 1A3 and 2B7 and has no significant effect on the pharmacokinetics of nirmatrelvir/ritonavir.

### 3.3. Case 3

The patient was only 20 days post-heart transplant when infected with SARS-CoV-2. SARS-CoV-2 invades cells through angiotensin-converting enzyme 2. The high expression of ACE2 in the cardiovascular system may allow the virus to directly damage the cardiovascular system, leading to disease progression, multiple organ failure, and even life-threatening conditions.^[14]^ The guidelines recommend^[7]^ that for organ transplant patients who may progress to severe COVID-19, nirmatrelvir/ritonavir antiviral therapy should be initiated as early as possible. The patient had recently undergone surgery, so the decision to abruptly discontinue immunosuppressants is questionable. The package insert indicates that tacrolimus is primarily metabolized by CYP3A4, with CYP3A5 playing a secondary role.^[2]^ When nirmatrelvir/ritonavir must be used concomitantly, 1/8 of the daily dose of tacrolimus should be administered on the first day, then discontinue it, and resume with 1/2 of the daily dose on the 6th day.^[8]^ Therefore, the clinical pharmacist suggested adjusting the tacrolimus dose to the minimum amount 3 days before starting nirmatrelvir/ritonavir treatment (0.5 mg, q12h), discontinued tacrolimus on the day of treatment initiation, continued administering mycophenolate mofetil (0.5 g, q12h), and closely monitored the progression of the disease at all times, identifying early signs of rejection, such as fatigue, malaise, loss of appetite, palpitations, fever, an unexplained decrease in BP, etc, as soon as possible.^[15]^ On the 4th day of nirmatrelvir/ritonavir treatment, the patient’s nucleic acid test for SARS-CoV-2 was negative, so nirmatrelvir/ritonavir was discontinued, and tacrolimus (1 mg, q12h) was restarted for antirejection therapy. Although the full 5-day course of nirmatrelvir/ritonavir recommended in the package insert was not completed, the patient’s temperature, blood test results, and lung CT scans showed improving trends in the later stages, indicating that the treatment was effective. The patient’s tacrolimus genetic testing result indicated a rapid metabolizer phenotype. Patients of this type are prone to rejection reactions due to insufficient drug concentrations. Therefore, clinical pharmacists recommend changing to cyclosporine and monitoring the blood drug concentration. The antihypertensive medication amlodipine besylate taken by the patient is metabolized by CYP3A4, and concomitant use with nirmatrelvir/ritonavir can increase the exposure of amlodipine by approximately 2-fold.^[2]^ Therefore, it was replaced with benidipine, which has no interaction. In addition, options for antihypertensive medications also include ACEIs such as enalapril, quinapril, and captopril; ARBs such as olmesartan, candesartan, and telmisartan; and hydrochlorothiazide or β-blockers.^[16]^ They have no significant interaction with nirmatrelvir/ritonavir and can be used safely. The patient experienced anxiety and fear during the treatment period and was unwilling to cooperate with the treatment. Benzodiazepine sedatives commonly used in critically ill patients, such as estazolam, when used in combination with nirmatrelvir/ritonavir, can lead to an increase in the blood concentration of the former, resulting in excessive sedation and respiratory depression. After consulting the relevant literature,^[17]^ the clinical pharmacist recommended the use of low-dose zolpidem tablets (5–10 mg, before bedtime) to improve patients’ sleep and mental state, which would be more conducive to the rehabilitation of patients with underlying diseases.

## 4. Discussion

COVID-19 is an acute infectious pneumonia that can affect multiple systems and organs simultaneously, including the cardiovascular system, liver, and kidneys.^[1]^ Patients who undergo heart transplantation have complex and variable conditions and are prone to developing severe COVID-19. Therefore, pharmaceutical care should be strengthened during the treatment process and address the main issues at different stages of the illness, such as antiviral treatment, infection control, and antirejection therapy, with appropriate interventions. The patient’s vital signs, laboratory tests, and imaging changes should be closely monitored, and the medication treatment plan should be adjusted in a timely manner.

In this paper, the use of immunosuppressants was adjusted for all 3 patients during hospitalization. Tacrolimus and cyclosporine are primarily metabolized by CYP3A4 and CYP3A5. Ritonavir, a potent inhibitor of CYP3A, can lead to elevated blood concentrations of these drugs, thereby increasing their toxicity levels. In this study, by adjusting the dosage of tacrolimus in advance, we successfully avoided the toxic reactions caused by elevated tacrolimus blood concentrations. In cases 1 and 2, immunosuppressants were discontinued for 3 days before initiating nirmatrelvir/ritonavir treatment. For Case 3, who underwent heart transplantation just 20 days prior, the pharmacist prioritized antirejection therapy by only discontinuing tacrolimus, while mycophenolate mofetil was used at a low dose throughout the entire course. None of the 3 patients progressed to critical COVID-19, and their tacrolimus and cyclosporine blood concentrations were within the normal range at discharge. Literature reports^[18]^ indicate that tacrolimus can cause acute kidney injury (AKI) in renal transplant recipients. Kulkarni et al^[19]^ compiled data from 18 studies on COVID-19 infection in liver transplant recipients and found that the incidence of secondary AKI following COVID-19 infection in liver transplant recipients was 33.22%. For case 2, due to severe renal insufficiency, the clinical pharmacist adjusted the nirmatrelvir/ritonavir dosage and strengthened renal function monitoring. For case 3, the antirejection medication was changed after genetic testing, resulting in a more complex treatment process. Both case 1 and case 2 experienced symptoms of abdominal pain. According to the literature, the main adverse reactions of nirmatrelvir/ritonavir include taste disturbance, diarrhea, hypertension, and myalgia.^[2]^ Moreover, for patients with a history of elevated triglycerides, vigilance should be exercised for the occurrence of pancreatitis.^[20]^ However, patients undergoing organ transplantation often require concurrent use of multiple medications, significantly increasing the risk of DDIs. For instance, when tacrolimus is co-administered with CYP3A4 enzyme inhibitors, such as triazole antifungal agents, its blood concentration can increase by 2-10-fold, posing a risk of acute nephrotoxicity. Conversely, co-administration with CYP3A4 enzyme inducers may result in subtherapeutic tacrolimus blood concentrations, potentially triggering rejection reactions. For patients after heart transplantation, we not only focus on DDIs involving immunosuppressive drugs but also assess their overall cardiovascular risk through multi-omics analysis. Based on this, we develop a comprehensive medication regimen that includes antihypertensive, lipid-lowering, and hypoglycemic agents. Specifically, we replace the CYP3A4-metabolized antihypertensive drug amlodipine besylate with benidipine, the lipid-lowering agent atorvastatin calcium tablets with pitavastatin calcium tablets, the corticosteroid prednisone with methylprednisolone, and the hypnotic drug estazolam with zolpidem. This multidimensional monitoring model effectively reduces therapeutic conflicts caused by drug interactions and improves patients’ long-term prognosis.

Combining the epidemiological characteristics of COVID-19, guidance from Chinese Experts on Prevention, Treatment Strategies, and Health Management of COVID-19 in the Organ Transplant Recipients (First Edition)^[21]^ and National Institutes of Health (NIH) guidelines,^[22]^ clinical pharmacists analyzed the key points of pharmaceutical care in the treatment process of HT patients: ① HT patients have a greater probability of developing severe COVID-19, and it is important to avoid further damage to cardiac function caused by hypoxia.^[23]^ ② HT patients have lower hepatic and renal function than normal individuals do, so attention should be given to substituting or adjusting medication doses to avoid dual-drug-induced injury.^[24]^ ③ Ritonavir, a component of nirmatrelvir/ritonavir, which is a strong CYP3A inhibitor, interacts with many immunosuppressants, necessitating individualized adjustments to the immunosuppressive regimen and close monitoring of blood drug concentrations, while weighing the pros and cons between antirejection and anti-infection therapies.^[25,26]^ ④ Paying attention to the interactions between nirmatrelvir/ritonavir and medications for underlying conditions, such as antihypertensive and lipid-lowering drugs, is recommended.^[27]^ ⑤ Be vigilant of gastrointestinal-related adverse reactions caused by nirmatrelvir/ritonavir, and is recommended to prophylactically administer probiotics to avoid secondary gastrointestinal infections.^[28]^ ⑥ When necessary, zolpidem can be administered to improve patients’ sleep and mental state, alleviate anxiety and tension,^[29]^ which is conducive to the treatment of COVID-19.

## 5. Conclusion

The main entry point for pharmacists to provide pharmaceutical services in the treatment of COVID-19 in HT patients is the combination of multiple medications and the long-term use of medications. Clinical pharmacists should thoroughly understand the patient’s past medication history and disease control status, review the relevant literature, promptly provide medication treatment plans and determine drug dosages to avoid further damage to underlying conditions caused by antiviral therapy. Moreover, individualized therapeutic drug monitoring should be conducted to combat the virus most effectively. Through reasonable and effective pharmaceutical care, the clinical cure rate of COVID-19 in patients after heart transplantation can be improved.

## Acknowledgments

We are grateful to Jun Su, Jing Zhang, Wei Yue for their technical support during the data collection process; and we appreciate the valuable comments provided by Professor Jinhua Chen on this study.

## Author contributions

**Conceptualization:** Lu Xu.

**Data curation:** Lu Xu, Xiongjie Yu.

**Funding acquisition:** Jing Zhang.

**Investigation:** Xiongjie Yu.

**Supervision:** Jun Su, Jinhua Chen.

**Validation:** Wei Yue.

**Visualization:** Jinhua Chen.

**Writing – original draft:** Lu Xu.
